# Exploration of the Links Between Psychosocial Well-being and Face Recognition Skills in a French-Speaking Sample

**DOI:** 10.5334/pb.1294

**Published:** 2024-09-03

**Authors:** Trishia Nigrou, Michel Hansenne, Christel Devue

**Affiliations:** 1 Psychology and Neuroscience of Cognition Research Unit, University of Liège, Belgium

**Keywords:** face recognition, individual differences, developmental prosopagnosia, social anxiety, depression, coping strategies, compensation strategies

## Abstract

Face recognition abilities vary tremendously in the general population. People at the lower end of the spectrum, those with developmental prosopagnosia, report stress, anxiety or social interaction issues due to their poor face recognition abilities. It is thus important to develop adequate diagnostic tools convenient to use for clinicians and to examine relationships between face recognition skills and negative affects. In the present study, we provide a validated French translation of the 20-item prosopagnosia index (PI20), a self-report measure used to detect people with developmental facial identity recognition deficits (Shah et al., 2015; Tsantani et al., 2021). We also examined links between face recognition skills measured with the PI20 and a standard face recognition test (Cambridge face memory test-CFMT; Duchaine & Nakayama, 2006) and measures of social anxiety (social interaction anxiety scale, social phobia scale) and negative affects (state trait anxiety scale, Beck depression inventory). We did not find any significant correlation between the CFMT and measures of psychosocial well-being and only found a weak positive association between the PI20 and social interaction anxiety. Although this association is weak and warrants further research, raising awareness about developmental face recognition issues may help improve the well-being of people with facial identity recognition deficits and provide new investigation or intervention avenues for clinicians who treat patients with social interaction anxiety.

The ability to recognise people varies widely in the general population (Bobak et al., 2016; Duchaine & Nakayama, 2006). People on the lower end of the spectrum present major face recognition issues that are not explained by neurological diseases or trauma—developmental prosopagnosia (Bate & Tree, 2017; but see Barton & Corrow, 2016, for a discussion of alternative views of the aetiology of developmental prosopagnosia). Face recognition issues can concern acquaintances, friends, or even family members and oneself (Kennerknecht et al., 2006). Since recognising others is paramount to successful social interactions, repeated failures at recognising or placing people could negatively impact people’s lives, creating stress, anxiety, or relationship difficulties.

Only a few studies have explored the psychosocial impact of face recognition issues. In qualitative studies, participants with developmental prosopagnosia associate their difficulties with stress, anxiety, or shame. They report avoidance or dependence on trusted relatives in social situations (Adams et al., 2019; Dalrymple et al., 2014; Yardley et al., 2008). Consistently, one correlational study on 138 participants drawn from the general population found a small negative association between performance on a standardised recognition test (i.e. the Cambridge Face Memory Test—CFMT; Duchaine & Nakayama, 2006) and social anxiety (Davis et al., 2011).

Further, while some developmental issues are widely known (e.g. dyslexia, autism), the public and clinicians are less aware of others like prosopagnosia (Kuvač Kraljević et al., 2022; de Lemos et al., 2022). Therefore, people with developmental prosopagnosia can appear rude or dismissive because of their recognition errors, aggravating their interactional difficulties. In fact, people can be unaware of their own difficulties late into adulthood (Murray et al., 2018) and many develop suboptimal compensatory strategies—relying on clothing or voice—without realising that others predominantly use faces (Barton & Corrow, 2016).

Standardised tools that are easy to use are thus necessary to continue exploring links between face recognition issues and other individual factors, but also to establish prevalence or compare populations. Self-report tests provide cheap and platform independent measures. They thus constitute good complement to existing computerised tests such as the CFMT or can serve as pre-screening measures before administrating those. While self-reported measures of face recognition issues exist in English (i.e. the 20-item Prosopagnosia Index—PI20; Shah et al., 2015), there is no such questionnaire available in French that is fast to administer (but see Palermo et al., 2017 in which a longer questionnaire—77-item— in French assessing metacognition about face recognition was used).

The objectives of this study were thus two pronged. First, we aimed at validating a French version of the PI20 (Shah et al., 2015). We expected PI20 scores to correlate with performance on the gold standard test, the CFMT (Duchaine & Nakayama, 2006) as shown previously (Gray et al., 2017; Matsuyoshi & Watanabe, 2021; Shah et al., 2015). Second, we explored relationships between face recognition abilities and psychosocial well-being measured via social anxiety and negative affects (i.e. anxiety and depression) questionnaires. We expected that if poor face recognition skills were associated with lower psychosocial well-being, it would most specifically concern anxiety linked to social interactions, as measured with the Social Interaction Anxiety Scale—SIAS (Davis et al., 2011; Mattick & Clarke, 1998).

## Method

### Participants

A priori power calculation indicated that to replicate the negative association (one-tailed test, as a specific direction is tested) between the CFMT and the SIAS found in an unselected sample (i.e. *r* = –0.177; Davis et al., 2011) with 0.80 power, we needed 193 participants. Further, since the PI20 consists of 20 items, a sample of 200 participants (i.e. 10 per item; Everitt, 1975) would be adequate to perform validation analyses. We thus recruited 217 French-speaking individuals via social media ads or the intranet of the University of Liège. Data of one participant who reported technical issues during the test were discarded. The final sample counted 216 participants (155 women, 57 men, 4 non-binary), aged between 18 and 80 years (Mean = 40.54 ± 14.11). A majority of participants (i.e. *N* = 207) reported being white and nine participants reported other ethnicities (i.e. 1 black, 2 latino/hispanic, 2 native American, 4 south Asian). The study was approved by the local ethics committee and participants gave informed consent before answering.

### Material

#### Face recognition measures

*CFMT*. This test measures the ability to learn and recognise new faces (Duchaine & Nakayama, 2006). Six faces are learned sequentially and on each test trial, participants attempt to recognise one learned face amongst three. After the initial learning phase, any six faces can appear amongst three on each trial. As the test progresses, viewing conditions departs more and more from learning conditions. The test consists of 72 trials. Higher scores represent better performance.

*PI20*. This self-reported measure taps into various aspects of face recognition difficulties (e.g. extent of issues, commission of specific errors, compensation strategies, or negative feelings). Participants rate their agreements with each statement on a 5-point Likert scale (1 = Totally disagree, 5 = Totally agree). Higher scores indicate more face recognition issues. Two authors, CD (French-English bilingual) and TN, first translated the original version into French independently before agreeing on a final version, see Table S1. In line with recent recommendations (Kunst & Bierwiaczonek, 2023), we used DeepL (https://www.deepl.com/) to produce a back translation from French into English. The back translation was identical in meaning to the original questionnaire, despite slight variations in phrasing (e.g. “poor memory” instead of “bad memory”).

#### Psychosocial well-being measures

*SIAS*. The scale consists of 20 items assessing anxiety specifically linked to social interactions—e.g. anxiety caused by being alone with one person or mixing up in a group (Mattick & Clarke, 1998). People rate how each statement is characteristic of themselves on a 5-point Likert scale (0 = not at all, 4 = extremely). The original questionnaire was translated in French by CD and TN with the same method as above. Cronbach’s α in our sample was 0.934, attesting of an excellent internal consistency.

*SPS*. The Social Phobia Scale (SPS) consists of 19 items targeting social phobia—e.g. fear of performing specific actions in front of others (Mattick & Clarke, 1998). Ratings are provided in the same way as for the SIAS. This questionnaire was also translated in French by CD and TN. Cronbach’s α in our sample was 0.932, again demonstrating an excellent internal consistency.

*STAI Y-A*. The Y-A subscale of the State-Trait Anxiety Inventory measures trait anxiety via 20 items (Spielberger, 1983). Ratings are scored from 1 (not at all) to 4 (very much so). We used a validated French translation of the original questionnaire (Bruchon-Schweitzer & Paulhan, 1990). Cronbach’s α in our sample was 0.925, indicating an excellent internal consistency.

*BDI*. The Beck Depression Inventory (BDI) consists of 21 items assessing depressive affects (Beck et al., 1961). Each statement has four possible answers presented on a scale between 0 and 3. We used a validated French translation of the BDI (Bourque & Beaudette, 1982). When participants give multiple answers to an item, the statement with the highest score is used to calculate the total score. Cronbach’s α in our sample was 0.874, showing good internal consistency.

### Procedure

The six assessments were administrated online via Testable (testable.org) in the following fixed order: PI20, SIAS, SPS, STAI-Y-A, BDI and CFMT.^1^ The PI20 and the CFMT were separated by the four other questionnaires in order to minimise any influence from filling out the PI20 onto the performance on the CFMT. Participants completed the test on their personal computers. They were asked to do so by themselves and in a quiet location. Before they began, they were also asked to calibrate their screen by adjusting a line on the screen to a payment (or other similar) card so as to standardise stimuli size.

## Results

Aggregated data are available at https://osf.io/cpxv3/.

### Validation of the French translation of the PI20

A reliability analysis indicated that our translated items had high internal consistency, Cronbach’s α = 0.942. Like in the original study (Shah et al., 2015), an exploratory factor analysis with Varimax rotation (parallel analysis based on principal components) conducted in JASP (JASP Team, 2023) suggested a single factor structure that accounted for 46.9% of the variance (vs. 61% in the original study). Items loadings ranged between 0.914 and 0.265 (Mean = 0.661, SD = 0.19; with all items between 0.914 and 0.4, except item 3). The way the one-factor model fitted the data was acceptable (RMSEA = 0.081, 90% CI [0.071–0.091]; TLI = 0.897).

Furthermore, Pearson’s correlation analysis showed a significant moderate association between scores on the PI20 and performance on the CFMT, *r* = –0.361, *p_one-tailed_* < 0.001, 95%CI [–0.471––0.239]. In other words, the lower people scored on a standard face recognition test, the more likely they were to report face recognition difficulties. The size of this correlation is consistent with results of previous studies (Gray et al., 2017; Matsuyoshi & Watanabe, 2021; Shah et al., 2015).

### Associations between face recognition skills and psychosocial well-being

Descriptive statistics for the six assessments administrated to our sample appear in Table 1. Out of 216 participants, 57 (i.e. 27.3%) had PI20 and/or CFMT scores that suggest face recognition issues (see details in “Group comparisons” below). We used correlational analyses to examine possible links between the two measures of face recognition abilities (i.e. PI20 and CFMT, respectively) and social anxiety and negative affects. Since the Shapiro-Wilk assumptions were violated for most pairs of measures, we used Spearman’s correlation analyses. As we had clear hypotheses about the directionality of the associations between measures of face recognition and psychosocial well-being measures (i.e. positive for the PI20 and negative for the CFMT), we used one-tailed tests. In order to balance Type I and Type II errors, and as we only conducted a small number of tests (i.e. four per measure of face recognition) and it seemed crucial to avoid Type II errors since well-being and care of people is potentially at stake, we followed Armstrong (2014)’s recommendations to not apply corrections for multiple comparisons.

**Table 1:** Means and ranges of scores on a standard face recognition test (CFMT) and self-report face recognition skills and psychosocial well-being questionnaires.


N = 216	CFMT	PI20	SIAS	SPS	STAI Y-A	BDI

Mean	0.754	48.9	30.3	20.1	45.9	34.4

Median	0.750	45.0	28.0	17.0	45.0	33.0

Standard deviation	0.133	16.6	15.9	14.9	11.0	8.46

Minimum	0.417	20	2	0	23	23

Maximum	1.00	90	69	70	73	69


*PI20*. As expected, there was a weak significant positive association between scores on the PI20 and the SIAS, *rho* = 0.179, *p_one-tailed_* = 0.004, 95%CI [0.069 – 1] but not between scores on the PI20 and the SPS, *rho* = 0.110, *p _one-tailed_* = 0.054, 95%CI [-0.003 – 1]. Scores on the PI20 were also weakly associated with trait anxiety, *rho* = 0.137, *p_one-tailed_* = 0.022, 95%CI [0.025 – 1], but not with depression, *rho* = 0.105, *p_one-tailed_* = 0.061, 95%CI [–0.007 – 1]. Results are shown in Table 2.

**Table 2:** Results of Spearman’s correlation analyses and partial correlations between scores on the PI20 and measures of social anxiety and negative affects.


	*rho*	*p*	95% CI LOWER	95% CI UPPER	*rho_partial_*	*P_PARTIAL_*

SIAS	0.179**	0.004	0.069	1	0.130*	0.029

SPS	0.110	0.054	–0.003	1	–0.052	0.776

STAI Y-A	0.137*	0.022	0.025	1	0.024	0.362

BDI	0.105	0.061	–0.007	1	0.026	0.350


*Note*. All tests are one-tailed for positive correlations. *rho_partial_* represents residual correlations between the PI20 and one other measure controlling for the other three measures. *p < 0.05, **p < 0.01.

Since measures of social anxiety and negative affects were all moderately to strongly correlated between themselves (see Table 3), we conducted partial correlation analyses to test associations between the PI20 and each measure while removing potential contributions from the other three measures. Results show that the weak association between the PI20 and the SIAS was the only one that remained significant, *rho* = 0.130, *p_one-tailed_* = 0.029.

**Table 3:** Results of Spearman’s correlation analyses between measures of social anxiety and negative affects.


	*rho*	95% CI LOWER	95% CI UPPER

SIAS–SPS	0.743***	0.677	0.798

SIAS–STAI Y-A	0.582***	0.487	0.664

SIAS–BDI	0.372***	0.251	0.482

SPS–STAI Y-A	0.585***	0.489	0.666

SPS–BDI	0.446***	0.332	0.547

STAI Y-A–BDI	0.717***	0.645	0.776


*Note*. ***p < 0.001.

*CFMT*. We did not replicate the weak negative association between CFMT scores and social interaction anxiety that was found previously (Davis et al., 2011). Indeed, performance on the CFMT did not significantly predict any of the psychosocial well-being measures, all *rho*s < |.130|, even when controlling for the impact of the other measures with partial correlation analyses, see Table 4.

**Table 4:** Results of Spearman’s correlation analyses and partial correlations between scores on the Cambridge Face Memory Test (CFMT) and measures of social anxiety and negative affects.


	*rho*	*p*	95% CI LOWER	95% CI UPPER	*rho_partial_*	*P_PARTIAL_*

SIAS	0.062	0.816	–1	0.173	–0.059	0.198

SPS	0.129	0.971	–1	0.238	0.129	0.970

STAI Y-A	0.061	0.813	–1	0.172	0.044	0.740

BDI	<0.001	0.504	–1	0.113	–0.075	0.138


*Note*. All tests are one-tailed for negative correlations. *rho_partia_*_l_ represents residual correlations between the CFMT and one other measure controlling for the other three measures.

*Group comparisons*. Finally, since CFMT may not necessarily capture various aspects of daily life performance with person identification (Devue et al., 2019) and since the ability of people within the normal range of face recognition skills to characterise their own skills is questionable (Tsantani et al., 2021), we split participants into two face recognition skills groups to compare their mean scores on the six tests. We intently used a liberal approach to ensure that the low skills group included all participants with potential difficulties. The face recognition issue (FRI) group (total *N* = 59) included the participants who scored below the CFMT cut-off (i.e. <58.4%) but had PI20 scores within the normal range (*N* = 13), those who reported mild to severe difficulties on the PI20 (i.e. scores >= 65) but with normal CFMT scores (*N* = 33) as well as participants who showed difficulties on both the PI20 and the CFMT (*N* = 13). The remaining participants (N = 157) were assigned to the control group. We hypothesised that CFMT and PI20 scores would differ between FRI participants and controls, even if people in the FRI group had various combinations of scores on both (for example, some people may have normal CFMT scores and problematic scores on the PI20 or vice versa).

Mann-Whitney tests for independent samples confirmed that CFMT scores were significantly higher in controls (Mean = 79.2%, *SD* = 11.1) than in FRI participants (Mean = 65.1%, *SD* = 13.1), *U*(214) = 2003, *p* < 0.001, *r* = 0.568. Likewise, PI20 scores were significantly lower in controls (Mean = 41.5, *SD* = 10.8) than in FRI participants (Mean = 68.6, *SD* = 13), *U*(214) = 585, *p* < 0.001, *r* = 0.874. However, the two groups’ mean social anxiety and negative affects scores did not differ significantly, all *p*s > 0.4.

## Discussion

This study provides a validated French translation of the PI20—a self-report questionnaire that measures face recognition difficulties—that has an excellent structural validity. This tool will be useful to identify people with potential face recognition issues in clinical or research contexts (Shah et al., 2015; Tsantani et al., 2021). In our unselected sample, performance on the PI20 was moderately correlated with performance on the most commonly used objective test of face recognition skills, the Cambridge Face Memory Test (Duchaine & Nakayama, 2006). The size of this relationship (i.e. rho = –.36) is consistent with previous findings (Gray et al., 2017; Matsuyoshi & Watanabe, 2021; Shah et al., 2015), and suggests, since the association is below 0.5, that although both tests may tap into common components of face processing, they do not measure the same processes (Abma et al., 2016).

We also show a weak but significant association between face recognition skills as measured with the PI20 and social anxiety measured with the Social Interaction Anxiety Scale (SIAS). This association is consistent with reports from people with developmental prosopagnosia (Adams et al., 2019; Dalrymple et al., 2014; Yardley et al., 2008) and with findings from one other quantitative study conducted on unselected samples (Davis et al., 2011). In contrast to another study that had found a significant association between CFMT scores and SIAS scores (Davis et al., 2011), we did not replicate that association. Further, we did not find any significant correlation between the CFMT, an objective measure of face recognition skills, and any of our psychosocial well-being measures. This was the case even when controlling for the potential contribution of other measures with partial correlation analyses, and despite adequate power and a moderate correlation between PI20 and CFMT scores. Therefore, the CFMT might not always be sensitive to daily life difficulties with faces, making possible associations between interaction anxiety and face recognition issues less obvious and reproducible. While self-report questionnaires such as the PI20 are not necessarily adequate to measure face recognition skills of people with normal or superior skills, it was shown to adequately identify people with deficient face recognition skills (Tsantani et al., 2021). The current study suggests that the PI20 may be more reliable than the CFMT in detecting associations that may exist between face recognition skills and anxiety. However, given the small size of this association, replication studies will be needed. Future studies could also assess whether personality factors (e.g. extraversion or neuroticism) may moderate the association between face recognition skills and social interaction anxiety.

Finally, we found no other reliable associations between face recognition skills as measured with the PI20 and the other negative affects assessed in the study (i.e. social phobia, trait anxiety or depression), as the weak association that existed between the PI20 and trait anxiety disappeared in partial analyses. This suggests a specific link between face recognition difficulties and social interaction anxiety. Of course, this does not mean that individuals with developmental prosopagnosia do not experience other forms of anxiety or negative affects linked to their poor face recognition abilities, as they report that they do during (semi-) structured interviews (Adams et al., 2019; Dalrymple et al., 2014; Yardley et al., 2008). This only suggests that associations between face recognition skills and negative affects are not obvious in unselected samples where people have virtually infinite other possible sources of negative affects.

While we cannot assume any causality from correlations, the link between face recognition skills and social interaction anxiety suggests that it may be relevant for clinicians who treat people for social anxiety issues to investigate whether these could be partly linked to face recognition difficulties. If so, educating patients unaware of their developmental face recognition issues or providing compensation strategies acknowledging these issues (e.g. preparing encounters or finding trusted people to get prompts from in social situations) could in some cases adequately complement interventions targeting social anxiety. The efficiency of different compensation strategies will need to be clarified in future research. For example, training with face recognition per se has very limited effects and does not generalise to untrained faces (Bate & Bennetts, 2014). In addition, participants in qualitative research claim that disclosing one’s face recognition issues has positive effects and mitigates negative judgments in the workplace but others voice concerns about negative impacts of disclosure (Adams et al., 2019). Since people report that their issues are not always believed or taken seriously, raising public awareness on developmental face recognition issues will also be an important step to support them.

In conclusion, this study provides a validated self-reported measure of face recognition skills in French and shows a weak positive association between that measure and social interaction anxiety. This association is weak and requires more research, including into possible personality trait moderators. It nevertheless suggests that raising awareness about developmental face recognition issues could improve the well-being of people with facial identity recognition deficits and interventions strategies of clinicians who treat patients with social interaction anxiety.

## Data Accessibility Statement

Aggregated data are available at https://osf.io/cpxv3/. We posted a draft of our manuscript on the preprint server PsyArXiv under the following URL https://osf.io/preprints/psyarxiv/4vbfc on 19 January 2024.

## Additional File

The additional file for this article can be found as follows:

10.5334/pb.1294.s1 Supplementary file. Table S1: Items of the French translation of the PI20 scale with means and standard deviations (in italics).

## Footnotes

While there is a possibility that the fixed order may have influenced responses to wellbeing questionnaires (e.g. if responding to the PI20 induced anxiety in people with poor face recognition skills), results suggest that this was not the case as scores on the PI20 did not correlate with scores on most wellbeing questionnaires.
