# Supplementary material for: Exploration of the Links Between Psychosocial Well-being and Face Recognition Skills in a French-Speaking Sample
Source: Psychol Belg. 2024 Sep 3;64(1):145–51. doi: 10.5334/pb.1294 (PMC11378709; doi:10.5334/pb.1294)
Supplement: Supplementary file. — Table S1: Items of the French translation of the PI20 scale with means and standard deviations (in italics). [file pb-64-1-1294-s1.pdf]

Table S1. Items of the French translation of the PI20 scale with means and standard deviations (in italics).

1	Mes capacités à reconnaitre les visages sont pires que celles de la plupart des gens.	2.62 *(1.34)*
2	J'ai toujours eu une mauvaise mémoire des visages.	2.63 *(1.37)*
3	Je remarque qu'il est plus facile de reconnaitre les gens qui ont des traits faciaux distinctifs.	3.88 *(1.06)*
4	Je prends souvent des personnes que j'ai déjà rencontrées pour des étrangers.	2.31 *(1.27)*
5	Quand j'étais à l'école, j'avais des difficultés à reconnaitre mes camarades de classe.	1.40 *(0.84)*
6	Quand les gens changent de coiffures ou portent des chapeaux, j'ai du mal à les reconnaitre.	2.37 *(1.20)*
7	Je dois parfois prévenir les nouvelles personnes que je rencontre que je suis « mauvais(e) avec les visages ».	2.20 *(1.50)*
8*	Je trouve qu'il est facile de visualiser des visages spécifiques dans mon esprit.	3.05 *(1.24)*
9*	Je suis meilleur(e) que la plupart des gens pour mettre un nom sur un visage.	3.68 *(1.18)*
10	Sans entendre la voix des personnes, j'ai des difficultés à les reconnaitre.	2.06 *(1.06)*
11	L'anxiété due à la reconnaissance des visages m'a mené(e) à éviter certaines situations sociales ou professionnelles.	1.74 *(1.07)*
12	Je dois fournir plus d'effort que les autres personnes pour mémoriser des visages.	2.44 *(1.39)*
13*	Je suis très confiant(e) en ma capacité à me reconnaitre sur des photos.	1.73 *(0.96)*
14	Je trouve parfois les films difficiles à suivre à cause de difficultés à reconnaitre les personnages.	2.31 *(1.37)*
15	Mes amis et ma famille pensent que j'ai une mauvaise reconnaissance des visages ou une mauvaise mémoire des visages.	2.06 *(1.31)*
16	J'ai l'impression de souvent offenser les gens en ne reconnaissant pas qui ils sont.	2.23 *(1.31)*
17*	Il m'est facile de reconnaitre des individus dans des situations qui obligent les personnes à porter des vêtements similaires (par exemple, des costumes, des uniformes, des maillots de bain).	3.05 *(1.25)*
18	Lors de réunions de famille, il m'arrive de confondre différents membres de ma famille entre eux.	1.27 *(0.69)*
19*	Je trouve facile de reconnaitre des célébrités sur des photos « avant qu'ils ne deviennent célèbres », même s'ils ont changé considérablement.	3.42 *(1.13)*
20	Il m'est difficile de reconnaitre des personnes familières quand je les rencontre hors contexte (par exemple, rencontrer un collègue de travail de façon inattendue en faisant du shopping).	2.46 *(1.30)*

Note. Asterisks indicate reverse scored items.
